# TiO_2_ Nanoparticles Dispersion in Block-Copolymer Aqueous Solutions: Nanoarchitectonics for Self-Assembly and Aggregation

**DOI:** 10.3390/jfb13020039

**Published:** 2022-04-09

**Authors:** Valeria Conti Nibali, Giovanna D’Angelo, Antonella Arena, Carmine Ciofi, Graziella Scandurra, Caterina Branca

**Affiliations:** 1 Department of Mathematical and Computer Science, Physical Sciences and Earth Sciences, University of Messina, Viale F. Stagno d’Alcontres, 98166 Messina, Italy; valeria.continibali1@unime.it (V.C.N.); gdangelo@unime.it (G.D.); 2 Department of Engineering, University of Messina, Contrada di Dio, I-98166, 98166 Messina, Italy; antonella.arena@unime.it (A.A.); carmine.ciofi@unime.it (C.C.); graziella.scandurra@unime.it (G.S.)

**Keywords:** pluronic F127, TiO_2_, self-assembly, dynamic light scattering

## Abstract

Achieving homogenous dispersion of nanoparticles inside a polymeric matrix is a great challenge for numerous applications. In the present study, we aim at understanding the role of different factors on the dispersion properties of TiO_2_ in pluronic F-127 mixtures. The mixtures were prepared with different pH and guest/host ratios and investigated by UV-Vis spectroscopy, dynamic light scattering, infrared spectroscopy and electrical conductivity. Depending on the preparation conditions, different amounts of TiO_2_ were loaded within the copolymer as quantitatively determined by UV-Vis spectroscopy. The different content of nanoparticles has direct implications on the gelation and micellization of pluronic analyzed by dynamic light scattering. The information derived on the self-assembly behavior was interpreted in relation to the infrared and conductivity measurements results. Together, these results shed light on the most favorable conditions for improving the nanoparticle dispersion inside the copolymer matrix and suggest a possible strategy to design functional nanoparticle-polymer systems.

## 1. Introduction

Nanoparticles exhibit unique properties that make them suitable for multifunctional applications. For most of them, effectively dispersing nanoparticles in solution is often necessary but not always easily achievable, and it requires a correct processing strategy. In some cases, the incorporation of nanoparticles within a polymer host became a winning strategy for homogeneous dispersions whose properties are a unique combination of the guest and the host. Tailoring these properties is a great challenge in designing materials with advanced performances.

Among transition metal oxide-based nanoparticles, titanium dioxide nanoparticles are the most extensively used in many different fields as gas sensors [1,2,3,4,5], solar cells [6], photocatalysts [7,8,9,10], and additives in food and cosmetics [11,12,13]. Moreover, thanks to their high biocompatibility and low toxicity, they have been extensively used in nanomedicine and nanobiotechnology for drug delivery, tissue engineering and others [14,15,16,17,18,19]. 

Whatever their field of application, the dispersion and stability of TiO_2_ nanoparticles in aqueous or in a more complex medium play a key role in improving the efficiency and performance of the systems. It is widely reported that the dispersion of nanoparticles in aqueous systems generally modifies the physicochemical properties, e.g., agglomeration state and surface charge variation [20]. Therefore characterizing the state of titania nanoparticles is of great significance, as they greatly affect many functionalities such as the photocatalytic activity [21,22], the toxicity [23], the particle electronic structure, surface defect density and the surface sorption sites [24].

Thus far, many studies have been performed to investigate the stability of TiO_2_ nanofluids with different concentrations and under various pH and ionic strength conditions [20,25,26,27,28,29,30]. The final objective of such research works is to define the best conditions for optimal performance of TiO_2_-based composites, whatever the field of application. In particular, there is a strong applicative interest in realizing well-ordered porous titania thin films. They have several advantages, such as a huge surface area and higher stability that significantly affect the efficiency of the electronic properties. Among various possible methods, the employment of structure-directing agents, including phosphates, ionic and nonionic surfactants, amines, and block copolymers [31,32,33,34,35,36,37,38,39,40,41,42], was very promising.

From these studies, it emerged that the structural and morphological properties of surfactant-based TiO_2_ systems greatly depend on the method of synthesis, the type and concentration of surfactant used, and the thermal treatment temperature other than the type and pH of copolymer solvent. Regardless of the procedure of synthesis used, as a final step, a high-temperature thermal treatment was performed, which enabled efficient removal of the copolymer template. Only after removal, the physical-chemical properties of the TiO_2_ films were investigated.

Unlike these studies, in the present work, our object of interest is the copolymer-TiO_2_ water dispersion as a whole. TiO_2_ nanoparticles were dispersed in pluronic F127/water suspensions at different copolymer concentrations and pH. 

Pluronic F127 is a nonionic triblock copolymer consisting of hydrophilic poly (ethylene oxide) and hydrophobic poly (propylene oxide) blocks (PEO–PPO–PEO) with a PEO/PPO ratio of 2:1. Above a critical micelle concentration, the block copolymers assemble into spherical micelles thanks to the difference in hydrophobicity between PEO and PPO blocks. Reversible gelation can occur only above some concentration and temperature [43,44,45,46,47,48,49,50,51].

The choice of pluronic F127 stems from its thermosensitivity and capability to solubilize and stabilize drugs inside the micelle core. This system is thus particularly interesting given the possible applications in many fields. In particular, it is one of the most widely used triblock copolymers in pharmaceutical formulations because of the good water solubility through the high content of EO, the low toxicity in the body and the ability to encapsulate any hydrophobic agents [52,53,54,55,56,57,58,59]. However, it is well known that the addition of co-solvents or solutes to pluronic water solutions can influence its properties, inducing phase changes [60,61,62,63,64,65,66,67,68]. 

In the present study, we are interested in analyzing how the dispersion or aggregation of titania nanoparticles inside pluronic F127 can influence the structural arrangements and, consequently, the systems’ dynamics. As it is well known, when nanoparticles are dispersed in solution, they undergo phenomena of agglomeration or aggregation, the difference being in the strength of interaction. Agglomerates are weakly bound collections of nanoparticles, whereas aggregates are tightly bound collections difficult to break up by mechanical forces. The classical Derjaguin–Landau–Verwey–Overbeek (DLVO) theory predicts the overall force between particles by combining Van der Waals attractive forces and repulsive forces arising from the electrical double layer (EDL) around the particles [69,70]. Other non-DLVO forces can influence nanoparticle dispersion, such as hydration, hydrophobic, steric, electronic, and electrostatic forces [69,70]. Among these, steric effects should be included in the case of nonionic polymer coatings; these are generally repulsive interactions, but if the polymer chains can form bridges between particles, that can cause aggregation. For all these reasons, it is clear that the dispersion properties are strongly dependent on parameters such as pH and ionic strength as they directly affect the zeta potential of the solution and the double layer thickness. 

Starting from these considerations, we have chosen to prepare the dispersions at pH values far from the isoelectric point (IEP) of the TiO_2_ suspension, approximately 6.2 [29]. In such a way, the presence of particle surface charge should enhance the electrostatic repulsion between metallic nanoparticles disfavoring, or suppressing, agglomeration. For the same reason, we decided to prepare the samples at a low ionic strength since, according to previous studies, the high ionic strength of the solution compresses the electrical double layer [20,29]. In preparing the pluronic-nanoparticles dispersions, another important factor to be considered is the copolymer concentration since, depending on temperature, it affects the phase behavior of the copolymer [43,44,45,46,47,48,49,50]. In the present case, the prepared base-pluronic solutions have weight fractions of 14% and 20 wt%; in the temperature range between 20 °C and 30 °C, the lowest concentration is always in a sol state, whereas the highest one move from a sol state to a gel-like state [50,51].

All these aspects were taken into consideration while we conducted our research with the final aim to define the best experimental conditions for optimal dispersion of TiO_2_ in pluronic F-127 mixtures. The samples, prepared under different experimental conditions, were investigated using UV-Vis spectroscopy, attenuated Fourier transform infrared spectroscopy (FTIR-ATR), dynamic light scattering (DLS) and electrical conductivity. The obtained results give valuable information on the dispersion state of nanoparticles; this knowledge is potentially useful for developing and optimizing copolymer-based nanosystems.

## 2. Materials and Methods

### 2.1. Materials

TiO_2_ nanoparticles dry powder anatase phase (average size 4–8 nm) were purchased from PlasmaChem GmbH (Berlin, Germany). The powder is free of organic stabilizer. Pluronic (F127, 12.600 Da) was obtained from Sigma-Aldrich (Dorset, UK). Hydrogen chloride (HCl), sodium hydroxide (NaOH), sodium chloride (NaCl) and deionized water were purchased from Sigma-Aldrich. All the chemicals are of analytical grade. 

### 2.2. Sample Preparation

TiO_2_ dry powder was suspended in 10 ml of solution at a 10.0 mg/mL concentration. The solutions had pre-adjusted pH values of 4 and 10 and an ionic strength equal to 0.001 M. The dispersions were magnetically stirred for 24 h and then sonicated for 15 min using a bath sonicator. After that, the suspensions were centrifuged for 30 min and 5 ml of each supernatant was sampled and analyzed by DLS. The size distribution calculated by using a CONTIN algorithm evidenced the presence of a bimodal distribution centered at 36 and 280 nm for the dispersion at pH 10 and 50 and 320 nm for the suspension at pH 4, respectively (data not shown). To remove the large agglomerates, the supernatants were then filtered and analyzed by UV-Vis spectroscopy to determine the effective TiO_2_ concentration. Absorption measurements were repeated in time for 24 h without observing any significant variation, thereby excluding any change in the dispersion state of TiO_2_ in this time range. 

Stock solutions of Pluronic F-127 at weight fractions of 14 wt% and 20 wt% were prepared, and aliquots of the TiO_2_ dispersions at the two pH values were then added. Attention was paid to adding the same TiO_2_ nanoparticle concentration in the final suspension (0.1 wt% corresponding to 1 mg/mL). 

Table 1 reports the sample labels as a function of the concentration of pluronic, TiO_2_ and pH conditions. The dispersions were gently stirred at 4 °C for 24 h and left overnight in the refrigerator until a clear solution was obtained. No sol-gel transition was observed for these systems up to 40 °C, as evidenced by the tube inversion method. The dispersions were stirred again and centrifuged; then, the supernatants were removed and transferred to fresh cuvettes for further analysis. No pH change from the stock TiO_2_ solutions was observed in the final dispersions. For comparison, two plain pluronic solutions with 14 wt% (PA) and 20 wt% (PB) concentrations were prepared. 

### 2.3. UV-Vis Spectroscopy

The UV-Vis measurements were made using a small-volume (100 μL) absorbance cuvette (Hellma 105.201-QS) and a UV-1700 Shimadzu spectrophotometer in the 280–700 nm range. All measurements were carried out at room temperature and replicated three times.

**Figure 1:**
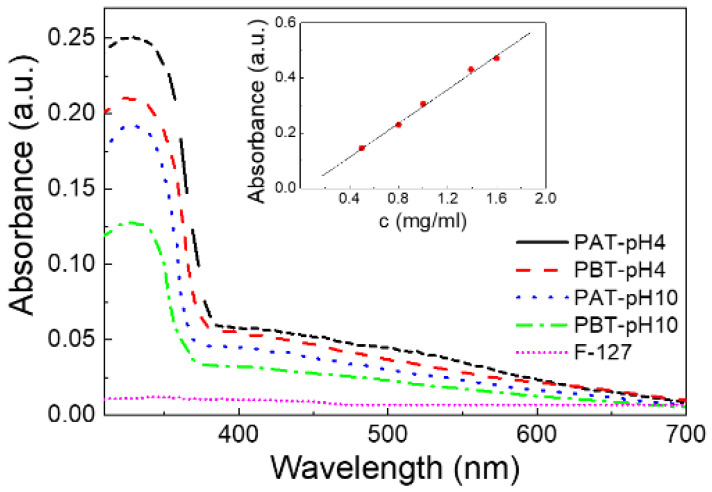
UV-Vis absorption spectra for the four pluronic-TiO_2_ dispersions studied in this work. For comparison, the absorption spectrum of a pluronic-F127 water solution (PA) is also reported. In the inset, the calibration plot for TiO_2_ is shown.

To quantitatively determine the concentration of TiO_2_ loaded in our samples, a calibration plot was first made by recording the UV-Vis spectra for five TiO_2_ water solutions of known concentration under the same pH and IS conditions. The intensity of the absorption peak at 330 nm was used to quantify TiO_2_. The linearity of the calibration curve was evaluated by linear regression analysis evidencing a high value of fitting degree (R^2^ > 0.999) (see the inset of Figure 1).

### 2.4. DLS Measurements

A laboratory-built goniometer equipped with single-mode fiber optics, two APD detectors, and a multi-τ digital time correlator (LS Instrument AG) ALV single-photon detector was used to perform DLS measurements. A He–Ne laser operating at 632.8 nm in linearly polarized single-frequency mode was used as an excitation source. The instrument is equipped with a thermostated recirculating bath which allows us to maintain the temperature with an accuracy of 0.1 °C. All measurements were performed at an angle of 90° to the incident beam at a temperature of 20 °C and 30 °C. Each sample was loaded in a square low-volume cuvette and thermalized at each temperature for 30 min before measuring. The repeatability of all measurements was verified with more than five measurements. From the DLS experiment, we obtained the normalized intensity auto-correlation function (ICF), *g*^2^ (*q*,*t*); details on the theoretical background of this technique are reported elsewhere [68,71,72,73]. Decay times are estimated from the ICFs according to a fit model reported in the results and discussion section. The CONTIN algorithm was also applied to obtain the size distribution for the TiO_2_ solutions. 

### 2.5. FTIR-ATR Spectroscopy

FTIR-ATR spectra covering the range 400–4000 cm^−1^ were recorded on a Bruker Vertex 80V FTIR spectrometer equipped with a Bruker Platinum ATR accessory with a single reflection diamond crystal. A background scan was recorded prior to the measurement and subtracted from the sample spectra. All spectra are the average of three independent measurements with 128 scans each, at a resolution of 2 cm^−1^. The ATR correction to each spectrum was applied using the OPUS software (Bruker optics). The spectra were normalized to the same area and compared to each other.

### 2.6. Electrical Conductivity 

The electrical conductivity values were obtained from electrical resistance measurements performed with an HP 4284a LCR meter. The instrument has been calibrated using measurements on liquids of known conductivity, and it has been ascertained that consistent measurements could be obtained in the 5 kHz–200 kHz frequency range. Measurements were performed by transferring, using a pipette, a small volume of the samples in ABS containers with a holding volume of 4.5 × 4.5 × 3 mm^3^ to limit the waste of material. The electrodes immersed in the samples were two small gold connectors 3.18 mm long and with a diameter of 0.5 mm, at a distance of 2.54 mm from each other. The conductivity values were obtained by comparing the resistance curves obtained for the samples with the calibration ones.

## 3. Results and Discussion

### 3.1. UV-Vis Spectroscopy 

The UV-Vis spectra were first analyzed for the quantitative assessment of TiO_2_ loaded into the pluronic-based dispersions. Figure 1 shows the results of the absorbance measurements for all the pluronic/TiO_2_ dispersions investigated and, as a reference, for a pluronic water solution (PA sample). For this latter sample, the absorbance spectrum does not reveal any significant absorption in the entire wavelength region investigated: the peak at λ = 330 nm, observed for all the pluronic/TiO_2_ samples, can be attributed exclusively to titanium dioxide. The observed changes in the intensity of this absorption peak suggest the presence of a higher loading of TiO_2_ nanoparticles in the suspensions under acidic conditions rather than in basic and, for the same pH, for the dispersions with the lowest pluronic concentration. 

Based on the calibration plot, see the inset of Figure 1, the effective concentration of TiO_2_ in the pluronic-based dispersions was estimated; the obtained results are reported in the last column of Table 1.

### 3.2. FTIR-ATR Measurements

**Figure 2:**
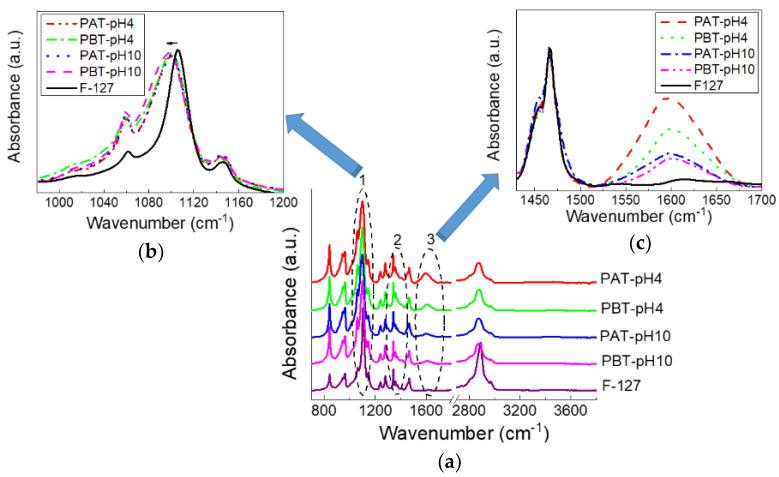
FTIR-ATR spectra for pluronic-F127 and pluronic-TiO_2_ dispersions in the dry state in the whole range 700–4000 cm^−1^(**a**). In (**b**,**c**), two magnified views in the range 980–1200 and 1430–1700 cm^−1^ are reported, respectively.

The effect of a different loading of TiO_2_ inside the investigated samples was also examined by FTIR-ATR spectroscopy. Measurements were performed on samples purged under dry nitrogen and left for equilibration for half an hour; nevertheless, as we will see, a certain amount of water is, in any case, retained within the samples. In Figure 2, the FTIR-ATR spectra of the TiO_2_-pluronic samples are shown and compared with the spectrum of pluronic F127. At a first glance, all the spectra appear very similar suggesting that the overall conformation of the copolymer does not change much. This is quite reasonable considering the very low quantity of TiO_2_ loaded into the samples. However, a more detailed inspection of the spectra revealed some changes that involve three specific bands (highlighted by numbered circles in the figure): the C–O–C stretching band (1050–1150 cm^−1^), labeled as 1, the CH_3_ symmetric deformation band (1370–1385 cm^−1^), labeled as 2 (both characteristic for pluronic) and the band around 1600 cm^−1^ (labeled as 3) characteristic for the bending mode of water Ti-OH. A magnified view for bands 1 and 3 are also shown in Figure 2. For all the samples, the C–O–C stretching mode observed for the anhydrous pluronic at 1106 cm^−1^ widens and shifts to a lower frequency, thereby suggesting the formation of more hydrogen bonds involving TiO_2_ and the oxygen atoms of the PEO chains. No significant change in peak frequency or bandwidth can be noticed among the pluronic-TiO_2_ samples for the same peak. On the contrary, clear changes can be seen for the bending mode at 1600 cm^−1^, whose intensity variation indicates the presence of different content of molecular water adsorbed on the TiO_2_ surface. This amount decreases following the TiO_2_ concentration as determined by UV-Vis absorption. It is also interesting to observe that, as noted in previous works [74,75], the intensity of the bending mode is significantly much higher than that of the OH stretching. This observation was attributed to an increased dipole moment due to interactions with the surface [74,75].

**Figure 3:**
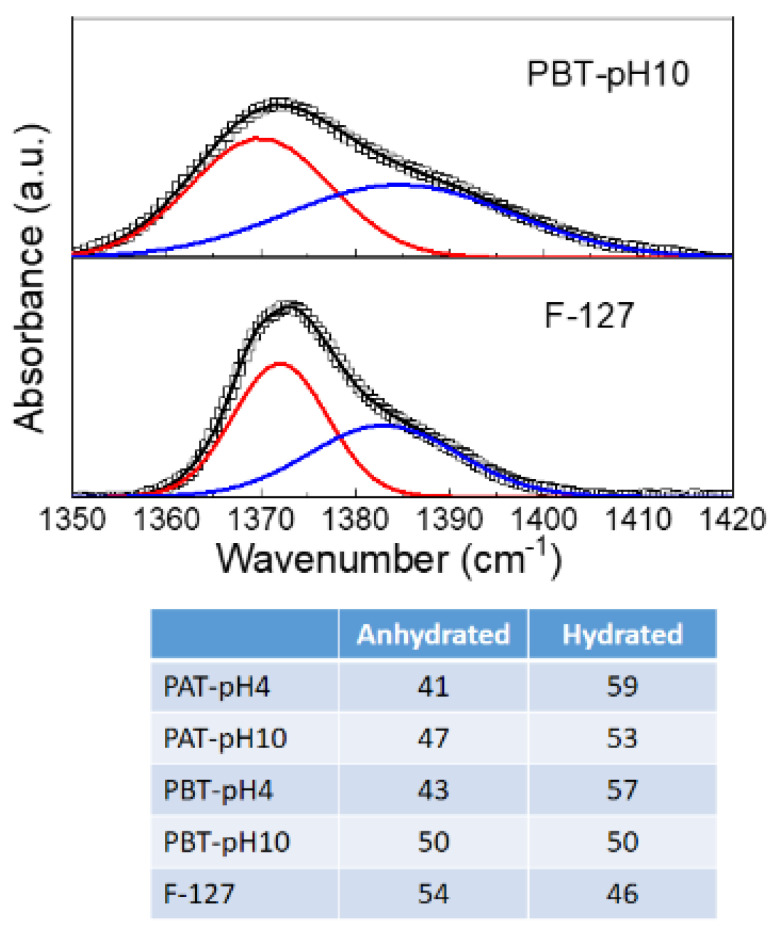
Representative deconvolution for dry pluronic F127 and PBT-pH10 into two Gaussian components representing the anhydrated (~1371 cm^−1^) and hydrated (~1383 cm^−1^) state of methyl groups. The experimental data are reported as squares, the best fit and the Gaussian components as continuous lines. Bottom: Integrated peak areas of the anhydrous and hydrated Gaussian component for all the samples investigated.

The CH_3_ symmetric band deserves a more detailed description. According to previous studies [67,76,77], this mode was analyzed using a Gaussian deconvolution method to separate the two components associated with anhydrous and hydrated methyl groups. For example, in Figure 2, the FTIR-ATR spectra for the dried PBT-pH10 and Pluronic F127 in the range from 1350 to 1420 cm^−1^ are shown with the corresponding best curving-fitting results. For clarity, all the other Gaussian components used to fit the spectral profiles in this region were subtracted. As one can see, in the presence of TiO_2,_ the symmetric deformation band broadens, indicating a higher structural disorder as just deduced from the C–O–C stretching mode. Besides this difference in the bandwidth, strong similarities are found among all the samples in the peak frequency for the two components that result centered at 1371 cm^−1^ (anhydrated component) and 1383 cm^−1^ (hydrated component). The integrated peak areas of these two vibrational modes were calculated from the fit parameters by normalizing the total area to 1. The percentage areas are reported in the table at the bottom of Figure 3. As reported in literature, the proportion of the anhydrous component increases with the dehydration of the micellar core. Thus it is strictly related to pluronic aggregation and micelle formation. As one can see, this hydrophobic effect weakens as the TiO_2_ content in the sample increases; in particular, for the PBT-pH10 sample, the micellization process is less disturbed by the presence of the nanoparticles; in the following, we will see that DLS evidence support this result.

### 3.3. DLS Measurements

**Figure 4:**
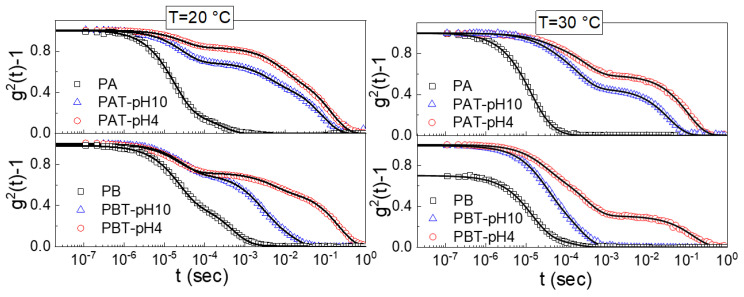
Normalized intensity autocorrelation functions (ICF) measured at a scattering angle of 90° for pluronic and pluronic-TiO_2_ solutions at T = 20 °C and T = 30 °C. The lines are the fits obtained with Equation (1).

Dynamic light scattering was performed to determine the different states of aggregation of titania-copolymer water dispersions. We analyzed the normalized autocorrelation functions (ICF) for all the prepared pluronic-TiO_2_ samples shown in Figure 4 for two different temperatures, 20 °C and 30 °C. The ICFs for the plane pluronic samples PA and PB are also shown for comparison. 

Two main findings are immediately evident from this figure; the first is the presence of a drastic drop in the *g*^2^ (*0*) for the PB pluronic solution at T = 30 °C. This decrease is a clear signature of a reduced mobility due to a transition from a sol to a gel state [50,51,68]. The absence of a similar drop for the other TiO_2_-pluronic suspensions suggests that the presence of the nanoparticles disturbs the gel formation, in agreement with what was observed by the inversion tube method.

The other characteristic that emerges is the quite complex profile of the normalized ICFs that can be accounted for by including multiple exponential-like terms. More precisely, as observed in previous studies [68], the ICFs for the pluronic aqueous solutions are described as a linear combination of an exponential function with a time scale *τ*_1_ and a stretched exponential function with a time scale *τ*_2_. For all the TiO_2_-pluronic samples, an additional long time stretched exponential with a time scale *τ*_3_ was required for a satisfactory fit. In the most general case, we used the following fitting function

(1) $$g^{\left(2\right)}\left(q,t\right)-1=\sigma_{1}^{2}\left\{A_{1}exp\left[-\left(t/\tau_{1}\right)\right]+A_{2}exp{\left[-\left(t/\tau_{2}\right)\right]}^{\beta_{2}}+A_{3}exp{\left[-\left(t/\tau_{3}\right)\right]}^{\beta_{3}}\right\}$$

where *σ*^2^_1_ is the initial amplitude of the ICFs, *A* measures the strength of the mode and *β* is the stretching exponent. As reported elsewhere [43,68], from the relaxation times. 

A correlation length *ξ* of the network can be estimated through the Stokes-Einstein relation, $\xi =kT/6\pi \eta q^{2}\tau$, where *k* is the Boltzmann constant, *η* the solvent viscosity and *q*^2^ the scattering vector.

According to the fit results, it was found that regardless of the pluronic concentration, temperature or pH, a fast relaxation occurs for all the samples on the time scale of 2.5·10^−5^–3.0·10^−5^ s. This fast decay seems to be a common characteristic of other pluronic-based nanoparticle systems. It has been ascribed to freely diffusing pluronic unimers with a radius of nearly 2 nm [43,44,45,46,47,48,49,50,51,68]. What profoundly differentiates the pluronic water systems from the pluronic-TiO_2_ ones is the population of unimers which is drastically reduced in the presence of TiO_2_. 

**Figure 5:**
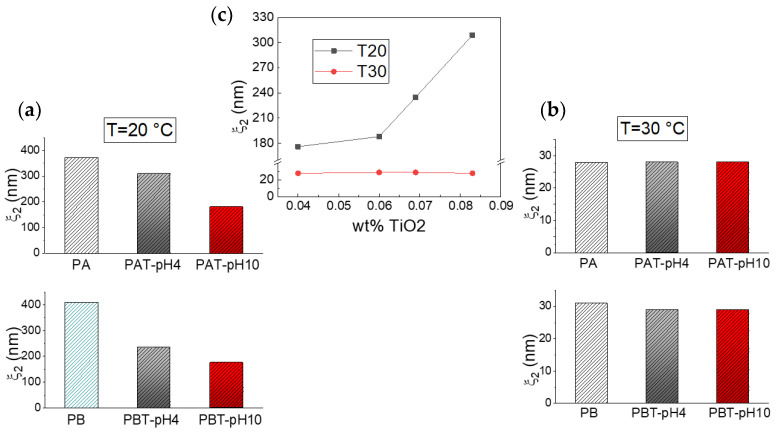
Correlation lengths *ξ*_2_ for the samples investigated at T = 20 °C (**a**) and T = 30 °C (**b**). The same data are reported in (**c**) as a function of the TiO_2_ concentration as determined by absorption spectroscopy.

Following the fast decay is a slower relaxation that we can observe on a time scale of ~2·10^−3^–5·10^−3^ s at a temperature of 20 °C and ~2·10^−4^–4·10^−4^ s at 30 °C. For pluronic solutions, this decay originates from the process of micellization, which implies, at low temperatures, an initial formation of large aggregates that, at higher temperatures, change into free micelles [43,68]. In the same time domain, a relaxation was also observed for all the pluronic-TiO_2_ samples at both temperatures, even if the presence of the TiO_2_ seems to determine a general slowing down for this dynamics. To better assess the influence of the nanoparticles on the assembly geometry, we analyzed the behavior of the correlation lengths *ξ*_2_, reported as histograms in Figure 5 (plots a and b) for two different temperatures. 

As cited above, the obtained data reproduce our previous findings for the pluronic solutions. Independently of pluronic concentration, this result confirms the formation of large aggregates at low T that decrease in size upon increasing temperature up to the typical size of micelles, 20–30 nm [68]. The same temperature trend in the size evolution can also be recognized for the pluronic-TiO_2_ samples but with some differences. It is evident that the presence of TiO_2_ hinders the formation of the large aggregates observed at the lowest temperature. More precisely, it is possible to state that this effect is more pronounced for those samples with the lowest TiO_2_ concentration. This is evident from Figure 5, plot c, where the same *ξ*_2_ data are reported as a function of the “real” TiO_2_ concentration, as determined by absorption spectroscopy. This suggests that the lower the TiO_2_ content, the better the metallic nanoparticles’ capability of being dispersed inside the network of PEO chains, hindering the formation of the large polymeric clusters. 

**Figure 6:**
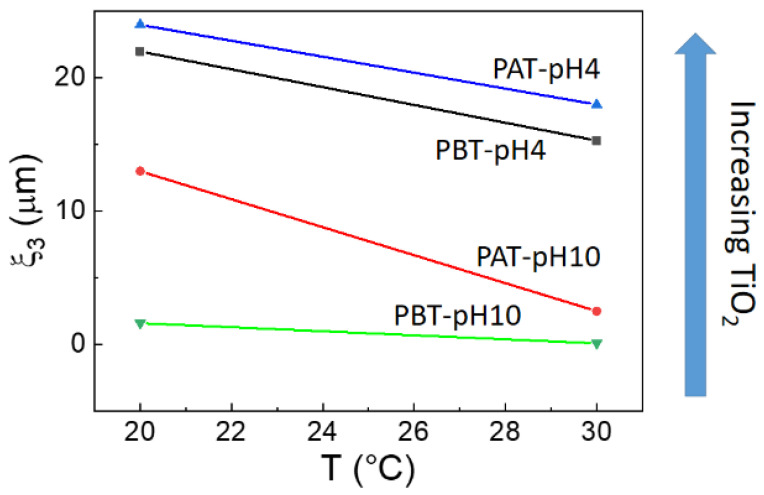
Correlation lengths *ξ*_3_ for the samples investigated as a function of temperature. The arrow shows the direction of the increment of TiO_2_ as determined by absorption spectroscopy.

This directly implies the slowest dynamic is occurring in the time range between 10^−3^ and 3·10^−1^ s. In fact, in this case, the corresponding correlation lengths *ξ*_3_, reported in Figure 6, evidence the presence of large micrometric aggregates that decrease in size with both increasing temperature and decreasing TiO_2_ content.

The last comment concerns the weight fractions for the intermediate and the slowest dynamics. From the evaluated fit parameters *A*_2_ and *A*_3_, it emerged that the presence of TiO_2_ determines a generally strong decrease in the percentage of micelles for all samples except for the PBT-pH10 one. This is the only one for which the percentage of micelles is comparable to the counterpart PB sample, thus confirming the infrared evidence. Moreover, for the same PBT-pH10 sample, the population of the micrometric aggregates, *A*_3_, is extremely low.

At this point, it remains to be clarified the nature of the micrometric aggregates. As stated in the introduction, at the pH values of this study, electrostatic repulsive interactions between titania nanoparticles are predominant. Consequently, the agglomeration of the nanoparticles due to a thickness reduction of the double layer should be unlikely. Anyway, electrical conductivity measurements helped us shed some light on this point.

### 3.4. Electrical Conductivity 

Thus far, many measurements of the electrical properties for water-based titania nanofluids or titania-polymer systems have been carried out [78,79,80], but not for dispersions of TiO_2_ inside a micellar matrix. Here, the interest in conductivity measurements arises from the possibility they offer to draw information on the nature of the micro-aggregates observed by DLS.

**Figure 7:**
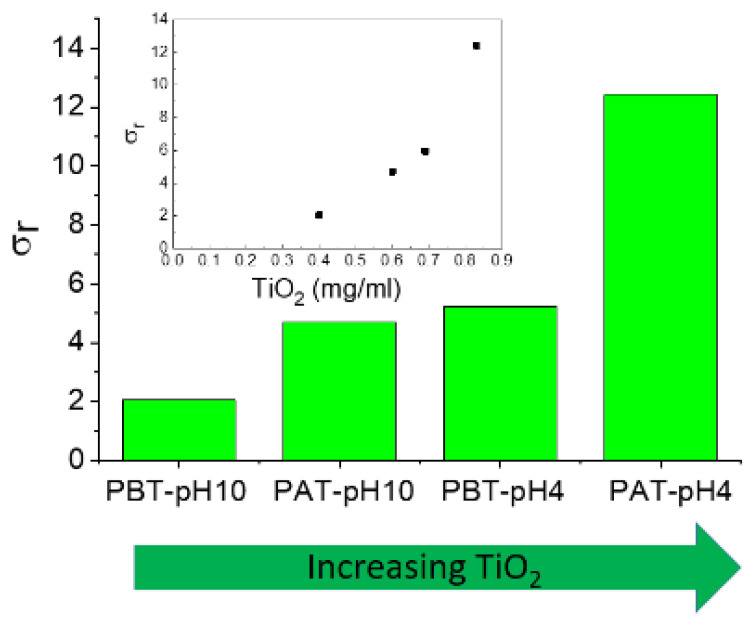
Relative electrical conductivity of the investigated samples. In the inset, the same data are reported as a function of the TiO_2_ concentration as determined by absorption spectroscopy.

The experimental data for the electrical conductivity of pluronic-TiO_2_ nanofluids are summarized in Figure 7, where we reported the relative electrical conductivity, *σ_r_*, defined as the ratio between the electrical conductivities of pluronic-TiO_2_ dispersions, *σ_n_*, and that of the base fluid, σ_f_, (pluronic solutions under the same conditions) *σ_r_ = σ_n_/σ_f_*. 

As expected, the addition of TiO_2_ to pluronic solutions increases the electrical conductivity of the systems. In principle, an increase in charge carriers determines an increase in effective electrical conductivity. Anyway, an increase in conducting nanoparticles could induce an increase in inter-particle collisions and consequently an enhancement of the probability of aggregation of the nanoparticles. This could determine a reduction in the effective number of charge carriers with a consequent deterioration of the effective charge transport. Even if the establishment of short conducting paths through aggregate-to-aggregate contact were assumed, the low intrinsic conductivity of TiO_2_ would exclude the possibility. In conclusion, the behavior of the electrical conductivity observed for our samples suggests that the presence of titania aggregates inside the polymeric matrix is unlikely.

## 4. Conclusions

The present paper investigated the properties of Pluronic F127 mixtures in the presence of TiO_2_ nanoparticles experimentally. Thus far, different copolymers have been used as a template for the formation of TiO_2_ mesostructures. Still, no study has examined the impact of the metallic nanoparticles on the self-assembly and aggregation behavior of the copolymer.

We have first analyzed the influence of pH and copolymer/nanoparticle ratio on the loading capacity of the copolymer. UV-Vis spectroscopy revealed that the maximum loading occurs at the lowest pluronic concentration under acidic conditions. Both FTIR-ATR and DLS analyses suggested that the different content of TiO_2_ inside the samples affects the micellization and aggregation of pluronic differently. In particular, it was observed that at the lowest TiO_2_ content, the formation of both nano and micrometric aggregates is less favored. Finally, general considerations on the nature of the electrostatic interactions at the pH values investigated together with the observed behavior of the electrical data allowed us to exclude the presence of single-species agglomerates. 

Therefore, we can hypothesize that the presence of the metallic nanoparticles favors the formation of an expanded network of micelles within which TiO_2_ is more or less homogeneously dispersed depending on its content. The fact that the optimal dispersion is obtained for the sample with the higher pluronic/TiO_2_ ratio corroborates the idea of TiO_2_ nanoparticles entrapped in a dense pluronic network. In future works, we plan to study the effects of TiO_2_ on different copolymers to develop the most appropriate strategy to design functional nanoparticle-copolymer systems.

## Figures and Tables

**Table 1:** Samples labels with the corresponding initial composition and pH values. In the last column, the amount of TiO_2_ loaded into the samples as determined by UV-Vis measurements is reported.

Sample	Pluronic wt%	TiO_2_ wt%	pH	TiO_2_ (Mean Value) mg/mL
PAT-pH4	14	0.1	4	0.83 (0.083 wt%)
PAT-pH10	14	0.1	10	0.69 (0.069 wt%)
PBT-pH4	20	0.1	4	0.60 (0.060 wt%)
PBT-pH10	20	0.1	10	0.40 (0.040 wt%)

## References

[1] Choi H., Sofranko A.C., Dionysiou D.D. (2006). Nanocrystalline TiO_2_ photocatalytic membranes with a hierarchical mesoporous multilayer structure: Synthesis, characterization, and multifunction. Adv. Funct. Mater..

[2] Chuangchote S., Jitputti J., Sagawa T., Yoshikawa S. (2009). Photocatalytic activity for hydrogen evolution of electrospun TiO_2_ nanofibers. ACS Appl. Mater. Interfaces.

[3] Kay A., Grätzel M. (1996). Low cost photovoltaic modules based on dye sensitized nanocrystalline titanium dioxide and carbon powder. Sol. Energy Mater. Sol. Cells.

[4] Arango A.C., Johnson L.R., Bliznyuk V.N., Schlesinger Z., Carter S.A., Hörhold H.-H. (2000). Efficient titanium oxide/conjugated polymer photovoltaics for solar energy conversion. Adv. Mater..

[5] Fujishima A., Rao T.N., Tryk D.A. (2000). Titanium dioxide photocatalysis. J. Photochem. Photobiol. C.

[6] Mor G.K., Shankar K., Paulose M., Varghese O.K., Grimes C.A. (2005). Enhanced photocleavage of water using titania nanotube arrays. Nano Lett..

[7] Fujishima A., Honda K. (1972). Electrochemical photolysis of water at a semiconductor electrode. Nature.

[8] Turchi C.S., Ollis D.F. (1990). Photocatalytic degradation of organic water contaminants: Mechanisms involving hydroxyl radical attack. J. Catal..

[9] Hoffmann M.R., Martin S.T., Choi W., Bahnemann D.W. (1995). Environmental applications of semiconductor photocatalysis. Chem. Rev..

[10] Pelizzetti E. (1995). Concluding remarks on heterogeneous solar photocatalysis. Sol. Energy Mater. Sol. Cells.

[11] Berardinelli A., Parisi F., Parrino F., Palmisano L. (2021). TiO_2_ in the food industry and cosmetics. Metal Oxides, Titanium Dioxide (Tio_2_) and Its Applications.

[12] Piccinno F., Gottschalk F., Seeger S., Nowack B. (2012). Industrial production quantities and uses of ten engineered nanomaterials in Europe and the world. J. Nanopart. Res..

[13] Weir A., Westerhoff P., Fabricius L., Hristovski K., von Goetz N. (2012). Titanium dioxide nanoparticles in food and personal care products. Environ. Sci. Technol..

[14] Wang Q., Huang J.-Y., Li H.-Q., Chen Z., Zhao A.Z.-J., Wang Y., Zhang K.-Q., Sun H.-T., Al-Deyab S.S., Lai Y.-K. (2016). TiO_2_ nanotube platforms for smart drug delivery: A review. Int. J. Nanomed..

[15] Park J., Cimpean A., Tesler A.B., Mazare A. (2021). Anodic TiO_2_ nanotubes: Tailoring Osteoinduction via Drug Delivery. Nanomaterials.

[16] Babitha S., Korrapati P.S. (2017). Biodegradable zein-polydopamine polymeric sca_old impregnated with TiO_2_ nanoparticles for skin tissue engineering. Biomed. Mater..

[17] Stan M.S., Nica I.C., Dinischiotu A., Varzaru E., Iordache O.G., Dumitrescu I., Popa M., Chifiriuc M.C., Pircalabioru G.G., Lazar V. (2016). Photocatalytic, antimicrobial and biocompatibility features of cotton knitcoated with Fe-N-Doped titanium dioxide nanoparticles. Materials.

[18] Seisenbaeva G.A., Fromell K., Vinogradov V.V., Terekhov A.N., Pakhomov A.V., Nilsson B., Ekdahl K.N., Vinogradov V.V., Kessler V.G. (2017). Dispersion of TiO_2_ nanoparticles improves burn wound healing and tissue regeneration through specific interaction with blood serum proteins. Sci. Rep..

[19] Hasan K.M.F., Horváth P.G., Alpár T. (2020). Potential Natural Fiber Polymeric Nanobiocomposites: A Review. Polymers.

[20] Jiang J., Oberdörster G., Biswas P. (2009). Characterization of size, surface charge, and agglomeration state of nanoparticle dispersions for toxicological studies. J. Nanopart. Res..

[21] Almquist C.B., Biswas P. (2002). Role of Synthesis Method and Particle Size of Nanostructured TiO_2_ on its Photoactivity. J. Catal..

[22] Sclafani A., Herrmann J.M. (1996). Comparison of the Photoelectronic and Photocatalytic Activities of various anatase and rutile forms of titania in pure liquid organic phases and in aqueous solutions. J. Phys. Chem..

[23] Jiang J., Oberdörster G., Elder A., Gelein R., Mercer P., Biswas P. (2008). Does nanoparticle activity depend upon size and crystal phase?. Nanotoxicology.

[24] Waychunas G.A., Kim C.S., Banfield J.F. (2005). Nanoparticulate Iron Oxide Minerals in soils and sediments: Unique properties and contaminant scavenging mechanisms. J. Nanopart. Res..

[25] Kaasalainen M., Aseyev V., von Haartman E., Şen Karaman D., Mäkilä E., Tenhu H., Rosenholm J., Salonen J. (2017). Size, Stability, and Porosity of Mesoporous Nanoparticles Characterized with Light Scattering. Nanoscale Res. Lett..

[26] Joo N.Y., Lee J., Kim S.J., Hong S.H., Park H.M., Yun W.S., Yoon M., Song N.W. (2013). Preparation of an aqueous suspension of stabilized TiO_2_ nanoparticles in primary particle form. J. Nanosci. Nanotechnol..

[27] Bielan Z., Dudziak S., Sulowska A., Pelczarski D., Ryl J., Zielińska-Jurek A. (2020). Preparation and Characterization of Defective TiO_2_. The Effect of the Reaction Environment on Titanium Vacancies Formation. Materials.

[28] Chakraborty S. (2019). An investigation on the long-term stability of TiO_2_ nanofluid. Mater. Today Proc..

[29] Suttiponparnit K., Jiang J., Sahu M., Suvachittanont S., Charinpanitkul T., Biswas P. (2011). Role of Surface Area, Primary Particle Size, and Crystal Phase on Titanium Dioxide Nanoparticle Dispersion Properties. Nanoscale Res Lett..

[30] Zhang X., Yin L., Tang M., Pu Y. (2010). Optimized method for preparation of TiO_2_ nanoparticles dispersion for biological study. J. Nanosci. Nanotechnol..

[31] Nilsson E., Furusho H., Terasaki O., Palmqvist A.E.C. (2011). Synthesis of nanoparticulate anatase and rutile crystallites at low temperatures in the Pluronic F127 microemulsion system. J. Mater. Res..

[32] Li Y.Q., Bastakoti B.P., Imura M., Hwang S.M., Sun Z.Q., Kim J.H., Dou S.X., Yamauchi Y. (2014). Synthesis of mesoporous TiO_2_/SiO_2_ hybrid films as an efficient photocatalyst by polymeric micelle assembly. Chem. Eur. J..

[33] Luo H., Wang C., Yan Y. (2003). Synthesis of mesostructured titania with controlled crystalline framework. Chem. Mater..

[34] Peng T., Zhao D., Dai K., Shi W., Hirao K. (2005). Synthesis of titanium dioxide nanoparticles with mesoporous anatase wall and high photocatalytic activity. J. Phys. Chem. B.

[35] Smarsly B., Grosso D., Brezesinski T., Pinna N., Boissiere C., Antonietti M., Sanchez C. (2008). Highly crystalline cubic mesoporous TiO_2_ with 10-nm pore diameter made with a new block copolymer template. Chem. Mater..

[36] Kim D.S., Han S.J., Kwak S.-Y. (2007). Synthesis and photocatalytic activity of mesoporous TiO_2_ with the surface area, crystallite size, and pore size. J. Colloid Interface Sci..

[37] Agarwala S., Ho G.W. (2009). Synthesis and tuning of ordering and crystallinity of mesoporous titanium dioxide film. Mater. Lett..

[38] Gajjela S.R., Ananthanarayanan K., Yap C., Gratzel M., Balaya P. (2010). Synthesis of mesoporous titanium dioxide by soft template based approach: Characterization and application in dye-sensitized solar cells. Energy Environ. Sci..

[39] Deng Y., Cai Y., Sun Z., Liu J., Liu C., Wei J., Li W., Liu C., Wang Y., Zhao D. (2010). Multifuntional mesoporous composite microsphere with well-designed nanostructure: A highly integrated catalyst system. J. Am. Chem. Soc..

[40] Samsudin E.M., Abd Hamid S.B., Juan J.C., Basirun W.J. (2015). Influence of triblock copolymer (pluronic F127) on enhancing the physico-chemical properties and photocatalytic response of mesoporous TiO_2_. Appl. Surf. Sci..

[41] Suwanchawalit C., Wongnawa S. (2010). Triblock copolymer-templated synthesis of porous TiO_2_ and its photocatalytic activity. J. Nanopart. Res..

[42] Roy S., Ghosh S.P., Pradhan D., Sahu P.K., Kar J.P. (2021). Morphological and electrical study of porous TiO_2_ films with various concentrations of Pluronic F-127 additive. J. Porous Mater..

[43] Brown W., Schillén K., Almgren M., Hvidt S., Bahadur P. (1991). Micelle and gel formation in a poly(ethylene oxide)/poly(propylene oxide)/poly(ethylene oxide) triblock copolymer in water solution: Dynamic and static light scattering and oscillatory shear measurements. J. Phys. Chem..

[44] Wanka G., Hoffmann H., Ulbricht W. (1994). Phase diagrams and aggregation behavior of poly(oxyethy1ene)-poly(oxypropylene)-poly(oxyethylene) triblock copolymers in aqueous solutions. Macromolecules.

[45] Alexandridis P., Hatton T.A. (1995). Poly(ethylene oxide)-poly(propylene oxide)-poly(ethylene oxide) block copolymer surfactants in aqueous solutions and at interfaces: Thermodynamics, structure, dynamics, and modeling. Colloids Surf. A.

[46] Prudhomme R.K., Wu G., Schneider D.K. (1996). Structure and rheology studies of poly(oxyethylene-oxypropylene-oxyethylene) aqueous solution. Langmuir.

[47] Malmsten M., Lindman B. (1992). Self-Assembly in Aqueous Block Copolymer Solutions. Macromolecules.

[48] Mortensen K. (1996). Structural studies of aqueous solutions of PEO–PPO–PEO triblock copolymers, their micellar aggregates and mesophases; a small-angle neutron scattering study. J. Phys. Condens. Matter.

[49] Alexandridis P., Holzwarth J.F., Hatton T.A. (1994). Micellization of Poly(ethylene oxide)-Poly(propylene oxide)-Poly(ethylene oxide) Triblock Copolymers in Aqueous Solutions: Thermodynamics of Copolymer Association. Macromolecules.

[50] Shaikhullina M., Khaliullina A., Gimatdinov R., Butakov A., Chernov V., Filippov A. (2020). NMR relaxation and self-diffusion in aqueous micellar gels of pluronic F-127. J. Mol. Liq..

[51] Chaibundit C., Ricardo N.M.P.S., Costa F.M.L.L., Yeates S.G., Booth C. (2007). Micellization and gelation of mixed copolymers P123 and F127 in aqueous solution. Langmuir.

[52] Batrakova E.V., Kabanov A.V. (2008). Pluronic block copolymers: Evolution of drug delivery concept from inert nanocarriers to biological response modifiers. J. Control. Release.

[53] Kulthe S.S., Inamdar N.N., Choudhari Y.M., Shirolikar S.M., Borde L.C., Mourya V.K. (2011). Mixed micelle formation with hydrophobic and hydrophilic Pluronic block copolymers: Implications for controlled and targeted drug delivery. Colloids Surf. B.

[54] Choi W.I., Lee J.H., Kim J.-Y., Kim J.-C., Kim Y.H., Tae G. (2012). Efficient skin permeation of soluble proteins via flexible and functional nano-carrier. J. Control. Release.

[55] Brunet-Maheu J.M., Fernandes J.C., De Lacerda C.A., Shi Q., Benderdour M., Lavigne P. (2009). Pluronic F-127 as a Cell Carrier for Bone Tissue Engineering. J. Biomater. Appl..

[56] Patel H.S., Shaikh S.J., Ray D., Aswal V.K., Vaidya F., Pathak C., Sharma R.K. (2022). Formulation, solubilization, and in vitro characterization of quercetin-incorporated mixed micelles of PEO-PPO-PEO block copolymers. Appl. Biochem. Biotechnol..

[57] Rahdar A., Hasanein P., Bilal M., Beyzaei H., Kyzas G.Z. (2021). Quercetin-loaded F127 nanomicelles: Antioxidant activity and protection against renal injury induced by gentamicin in rats. Life Sci..

[58] Kassa S.B., Taslimi P., Özel S., Gür B., Gülçin I., Onganer Y. (2022). Effects of some phenolic compounds on the inhibition of α-glycosidase enzyme-immobilized on Pluronic^®^F127 micelles: An in vitro and in silico study. Colloid Surf. A.

[59] Rahdar A., Hajinezhad M.R., Barani M., Sargazi S., Zaboli M., Ghazy E., Baino F., Cucchiarini M., Bilal M., Pandey S. (2022). Pluronic F127/Doxorubicin microemulsions: Preparation, characterization, and toxicity evaluations. J. Mol. Liq..

[60] Jiang J., Li C., Lombardi J., Colby R.H., Rigas B., Rafailovich M.H., Sokolov J.C. (2008). The effect of physiologically relevant additives on the rheological properties of concentrated Pluronic copolymer gels. Polymer.

[61] Pradines B., Djabourov M., Vauthier C., Loiseau P.M., Ponchel G., Bouchemal K. (2015). Gelation and micellization behaviors of pluronic^®^F127 hydrogel containing poly(isobutylcyanoacrylate) nanoparticles specifically designed for mucosal application. Colloids Surf. B.

[62] Dey J., Kumar S., Nath S., Ganguly R., Aswal V.K., Ismail K. (2014). Additive induced core and corona specific dehydration and ensuing growth and interaction of Pluronic F127 micelles. J. Colloid Interface Sci..

[63] Nelson A., Cosgrove T. (2005). Small-Angle Neutron Scattering Study of Adsorbed Pluronic Tri-Block Copolymers on Laponite. Langmuir.

[64] Zhang W., Gilstrap K., Wu L., Bahadur R., Moss M.A., Wang Q., Lu X., He X. (2010). Synthesis and characterization of thermally responsive pluronic F127-chitosan nanocapsules for controlled release and intracellular delivery of small molecules. ACS Nano.

[65] Perry C., Hebraud P., Gernigon V., Brochon C., Lapp A., Lindner P., Schlatter G. (2011). Pluronic and β-cyclodextrin in water: From swollen micelles to self-assembled crystalline platelets. Soft Matter.

[66] Nambam J.S., Philip J. (2012). Effects of Interaction of Ionic and Nonionic Surfactants on Self-Assembly of PEO−PPO−PEO Triblock Copolymer in Aqueous Solution. J. Phys. Chem. B.

[67] Branca C., Khouzami K., Wanderlingh U., D’Angelo G. (2018). Effect of intercalated chitosan/clay nanostructures on concentrated pluronic F127 solution: A FTIR-ATR, DSC and rheological study. J. Colloid Interface Sci..

[68] Branca C., D’Angelo G. (2019). Aggregation behavior of Pluronic F127 solutions in presence of chitosan/clay nanocomposites examined by dynamic light scattering. J. Colloid Interface Sci..

[69] Derjaguin B.V., Landau L.D. (1941). Theory of the stability of strongly charged lyophobic sols and of the adhesion of strongly charged particles in solutions of electrolytes. Progr. Surf. Sci..

[70] Shrestha S., Wang B., Dutta P. (2020). Nanoparticle processing: Understanding and controlling aggregation. Adv. Colloid Interface Sci..

[71] Branca C., Wanderlingh U., D’Angelo G., Crupi C., Rifici S. (2015). Study of the dynamical behavior of sodium alginate/myoglobin aqueous solutions: A dynamic light scattering study. J. Mol. Liq..

[72] Hassan P.A., Rana S., Verma G. (2015). Making sense of brownian motion: Colloid characterization by dynamic light scattering. Langmuir.

[73] Berne B.J., Pecora R. (1975). Dynamic Light Scattering: With Applications to Chemistry, Biology, and Physics.

[74] Finnie K.M., Cassidy D.J., Bartlett J.R., Woolfrey J.L. (2001). IR Spectroscopy of Surface Water and Hydroxyl Species on Nanocrystalline TiO_2_ Films. Langmuir.

[75] Bedurftig K., Volkening S., Wang Y., Wintterlin J., Jacobi K., Ertl G. (1999). Vibrational and structural properties of OH adsorbed on Pt(111). J. Chem. Phys..

[76] Su Y.L., Wang J., Liu H.Z. (2002). FTIR spectroscopic investigation of effects of temperature and concentration on PEO-PPO-PEO block copolymer properties in aqueous solutions. Macromolecules.

[77] Su Y.L., Wang J., Liu H.Z. (2002). Melt, hydration, and micellization of the PEO–PPO–PEO block copolymer studied by FTIR spectroscopy. J. Colloids Interface Sci..

[78] Islam M.R., Shabani B., Rosengarten G. (2017). Electrical and Thermal Conductivities of 50/50 Water-ethylene Glycol Based TiO_2_ Nanofluids to be Used as Coolants in PEM Fuel Cells. Energy Procedia.

[79] Chereches E.I., Minea A.A. (2019). Electrical Conductivity of New Nanoparticle Enhanced Fluids: An Experimental Study. Nanomaterials.

[80] Sikdar S., Basu S., Ganguly S. (2011). Investigation of electrical conductivity of titanium dioxide nanofluids. Int. J. Nanopart..

